# R Peak Detection Method Using Wavelet Transform and Modified Shannon Energy Envelope

**DOI:** 10.1155/2017/4901017

**Published:** 2017-07-05

**Authors:** Jeong-Seon Park, Sang-Woong Lee, Unsang Park

**Affiliations:** ^1^Department of Multimedia, Chonnam National University, 50 Daehak-ro, Yeosu, Jeollanamdo 59626, Republic of Korea; ^2^Department of Software, Gachon University, 1342 Seongnam-daero, Sujeong-gu, Seongnam, Gyeonggido 13120, Republic of Korea; ^3^Department of Computer Science & Engineering, Sogang University, 35 Baekbeom-ro, Mapo-gu, Seoul 04107, Republic of Korea

## Abstract

Rapid automatic detection of the fiducial points—namely, the P wave, QRS complex, and T wave—is necessary for early detection of cardiovascular diseases (CVDs). In this paper, we present an R peak detection method using the wavelet transform (WT) and a modified Shannon energy envelope (SEE) for rapid ECG analysis. The proposed WTSEE algorithm performs a wavelet transform to reduce the size and noise of ECG signals and creates SEE after first-order differentiation and amplitude normalization. Subsequently, the peak energy envelope (PEE) is extracted from the SEE. Then, R peaks are estimated from the PEE, and the estimated peaks are adjusted from the input ECG. Finally, the algorithm generates the final R features by validating R-R intervals and updating the extracted R peaks. The proposed R peak detection method was validated using 48 first-channel ECG records of the MIT-BIH arrhythmia database with a sensitivity of 99.93%, positive predictability of 99.91%, detection error rate of 0.16%, and accuracy of 99.84%. Considering the high detection accuracy and fast processing speed due to the wavelet transform applied before calculating SEE, the proposed method is highly effective for real-time applications in early detection of CVDs.

## 1. Introduction

An electrocardiogram (ECG) is a recording of the electrical activity of the heart [1, 2] and a graphical representation of the signals obtained from electrodes placed on the skin near the heart [1, 3, 4]. The recent use of computers in conducting ECG analysis allows the patterns of the ECG signal, composed of multiple cycles that include numerous sample points, to be visualized [5]. Some of these sample points are fiducial—namely, the P wave, QRS complex, and T wave [4]. The identification of these points is a critical step in analyzing the ECG signal and has become possible by analyzing its morphological patterns [6].

**Figure 1:**
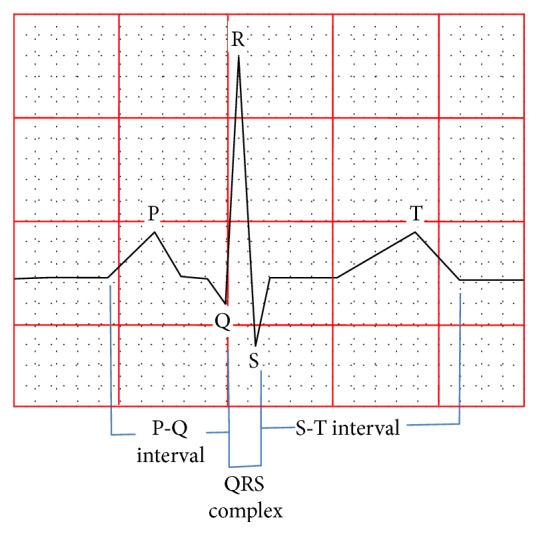
Fiducial points of an ECG signal: P wave, QRS complex, T wave, and time intervals.

In 2012, cardiovascular diseases (CVDs) accounted for 37% of premature (under the age of 70) noncommunicable disease mortality [7–9]. The detection of fiducial points is critical for the initial diagnosis and analysis of CVDs. In Figure 1(), we can see the peaks of the QRS complex, the highest of which is known as the R peak in the QRS interval [10]. Other time segments are also shown, such as the P-Q and S-T segments. The R peak in the QRS interval is the most important feature for analyzing the ECG data. All these waves are electrical manifestations of the contractile activity of the heart [11]. Detection of the main characteristic waves in an ECG is one of the most essential tasks, and the performance of any CVD analysis method depends on the reliable detection of these waves. R peak detection in ECG is one such method that is widely used to diagnose heart rhythm irregularities and estimate heart-rate variability (HRV) [12, 13].

Significant research efforts have been devoted to the detection of the fiducial points of an ECG signal. Those methods include slope-based threshold methods [14, 15], wavelet-transform-based methods [1, 4, 10, 16–18], mathematical-morphology-based methods [6, 19], digital filtering methods [14, 20, 21], and Shannon energy envelope- (SEE-) based methods [22–25]. There are also other studies that use the wavelet transform for denoising ECG signals [4, 17].

The Pan and Tompkins methods (PT) [14] appear to be the most common benchmark given that they incorporate several fundamental techniques including low-pass filtering, high-pass filtering, derivative filtering, squaring, and windowing for the detection of the R peaks. The principal drawback of a filtering-based approach is the adverse effect on performance [20] because of the change in frequency of the characteristic wave. Shannon energy with the Hilbert transform method (SEHT) [22] provides good accuracy for detecting R peaks. However, the Hilbert transform in SEHT requires large memory and processing time, making it unsuitable for real-time application. Moreover, SEHT detects many noise peaks in ECG data with long pauses. Zhu and Dong [23] developed an R peak detection method called PSEE by using only the SEE. The authors used an amplitude threshold that affects the performance of the algorithm for valid peak detection. A QRS complex generally overlaps in the frequency domain [26], resulting in false positive detections. Some of the threshold techniques are highly noise-sensitive [17]; therefore, developments of sophisticated, automatic, and computationally efficient techniques are required that can outperform existing methods to ensure the real-time analysis of an ECG for the proper diagnosis of CVDs.

In this paper, we propose a novel approach of using the wavelet transform (WT) and modified SEE for the rapid detection of R peaks in the QRS complex. First, the proposed WTSEE algorithm performs a WT to reduce the size and noise of ECG signals and subsequently calculates the SEE after first-order differentiation and amplitude normalization. Following this step, the peak energy envelope (PEE) is made from the SEE for easy identification of peaks. R peaks are then estimated from the PEE, and the estimated peaks are adjusted from the input ECG. Finally, the algorithm generates the final R peaks by validating R-R intervals and updating the extracted R peaks.

The proposed R peak detection method was validated using 48 first-channel ECG records of the MIT-BIH arrhythmia database with sensitivity, positive predictability, detection error rate, and accuracy. We also measured the mean of R-R interval (MRR), standard deviation of normal to normal R-R intervals (SDNN), and root mean square of successive heartbeat interval differences (RMSSDs), which can be used for analyzing heart rate variability.

The remainder of this paper is organized as follows. In Section 2, we discuss the proposed R peak detection method using the wavelet transform and SEE. In Section 3, we provide the experimental results, the performance analysis of the proposed method, and a discussion of the real-time implementation. In Section 4, we conclude our work and provide insight into future study.

## 2. Methods

An ECG signal consists of many cycles of P, Q, R, and S waves, with each cycle comprising many sample points. In MIT-BIH record 100, a cycle comprises approximately 280 sample points [27]. There are up to 10 fiducial points that determine the overall characteristics of a cycle of ECG signal. Among various existing SEE-based methods, band-pass filters, such as the Chebyshev type I filter and Butterworth filter, are used for denoising input ECG signals as preprocessing steps. In the proposed method, wavelet transform replaces the band-pass filters by level 2 down-sampling, soft thresholding for denoising detailed coefficients [17], and reconstructing the level 1 signal. By applying this procedure, we can reduce the size and time required for extracting R peaks.

**Figure 2:**
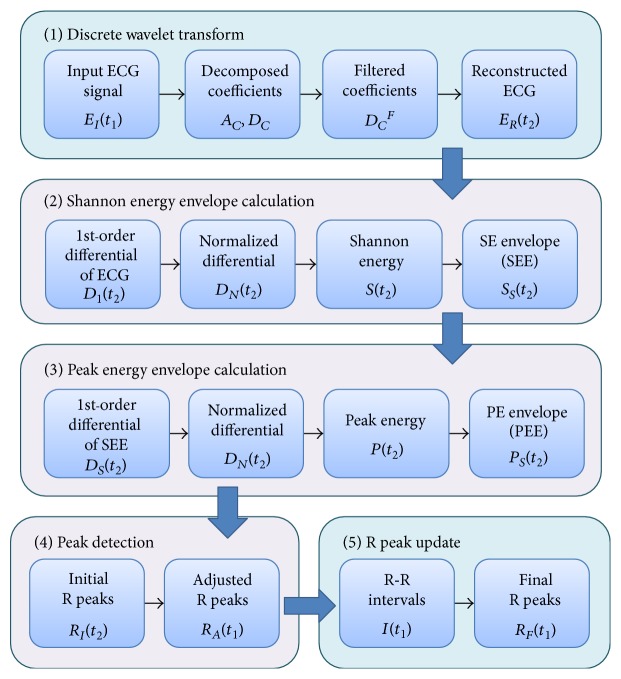
Data flow in the proposed R peak detection method using WT and modified SEE.

The proposed WTSEE algorithm extracts these fiducial points (R peaks) from the down-sampled ECG signals by calculating SEE, repeating a similar procedure as SEE to emphasize peak information (thus, we call this procedure the peak energy envelope), detecting R peaks, and updating the R peaks by comparing the R-R intervals.  Figure 2 presents the data flow in the proposed R peak detection algorithm with its principle stages. First, the proposed WTSEE algorithm performs a wavelet transform, instead of a band-pass filter, to reduce the size and noise of ECG signals. It then calculates SEE after first-order differentiation and amplitude normalization. In the third stage, the PEE is created from the SEE for easy identification of peaks. In the next stage, R peaks are estimated from the PEE, and the estimated peaks are adjusted from the input ECG. Finally, the algorithm generates the final R peaks by validating R-R intervals and updating the extracted R peaks. In Figure 2, original time space and down-sampled time space are represented by *t*_1_ and *t*_2_, respectively.

### 2.1. Discrete Wavelet Transform

**Figure 3:**
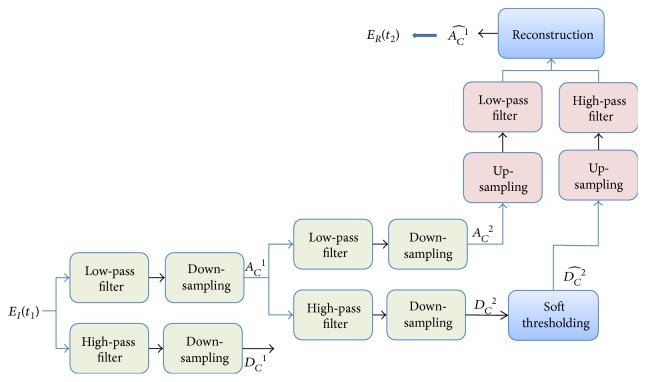
Procedure of wavelet transform comprising filtering, down-sampling, thresholding, up-sampling, and reconstruction.

The wavelet transform (WT) is a good technique for signal compression and noise reduction. The computational complexity for the discrete wavelet transform (DWT) is *O*(*n*). Wavelet analysis combines filtering and down-sampling as shown in Figure 3 [4, 17].

(1) As the first step of the proposed method, WT reduces the size and noise of an ECG. By applying these transform, memory requirements and processing time are dramatically reduced.

**Figure 4:**
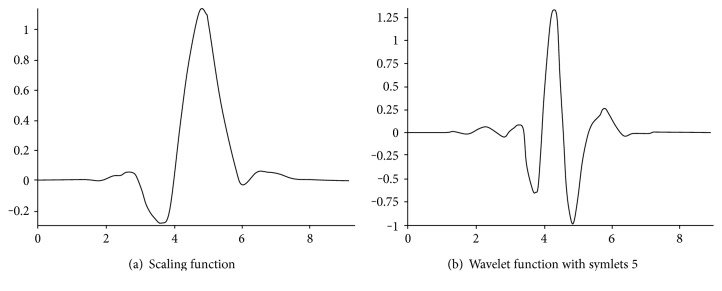
Comparison of scaling function and wavelet function with symlets 5 [28].

Symlets wavelet (sym5) is chosen as the wavelet function to decompose the ECG signals, and the thresholding method is employed to remove the noise [4].  Figure 4 demonstrates the difference between the scaling function and wavelet function with symlets [28]. By applying this procedure, the peak signal becomes clearer. The sym5 wavelet transformation is performed as

(1) $$\begin{array}{c} \left[A_{C},D_{C}\right]=dwt\left(E_{I},{}^{'}\mathrm{sym}5^{'}\right), \end{array}$$

where *A*_*C*_ is an approximation coefficient vector, *D*_*C*_ is a detail coefficient vector, *E*_*I*_ is an input ECG signal vector, and *dwt*(^∗^) is a DWT function.

(2) To reduce the noise of an ECG signal, we applied a soft thresholding that is recognized as more powerful than hard thresholding as

(2) DCj^=signDCj ∣ DCj ∣ −t, ∣ DCj ∣ >t0,    ∣ DCj ∣ ≤t,

where coefficients DCj^ and *D*_*C*_(*j*) are detail coefficients after and before thresholding, respectively.

In the proposed scheme, we chose the universal threshold selection method. Here, the value of threshold (*t*) is computed as

(3) $$\begin{array}{c} t=\sigma \sqrt{\frac{2log\left(N\right)}{N}}, \end{array}$$

where *N* is the total number of wavelet coefficients and σ = median(|*D*_*C*_(*j*)|)/0.6745 is the standard deviation of the noise.

After the noise removal, reconstruction is applied to obtain noise-free ECG signal as follows:

(4) EF=idwtAC,DC ^,  'sym5',

where *A*_*C*_ is a previously extracted approximation coefficients vector, DC ^ is a noise-removed detail coefficient vector, *E*^*F*^ is a denoised ECG signal, and *idwt*(^∗^) is an inverse DWT function.

### 2.2. Shannon Energy Envelope Calculation

(3) After applying the 2nd DWT along with noise removal and reconstruction, we perform the first-order differentiation of the signal to obtain the slope information. The first-order differentiation is equivalent with a high-pass filter that passes high-frequency components (QRS complex) and attenuates lower frequency components (P and T waves). The mathematical implementation of the first-order differentiation can be shown as

(5) $$\begin{array}{c} D_{n}=E_{n+1}^{F}-E_{n}^{F}. \end{array}$$

(4) Next, the signal is normalized to scale its value to 1. This is to prepare the signal for SEE computation.

(6) Dn^=Dnmax ∣ Dn ∣ .

(5) After differentiating the ECG signal, it becomes a bipolar signal. Given that the method is based on peak detection, we must transform the differentiated signal into a unipolar signal. The unipolar signal can be obtained by the following Shannon energy function:

(7) SnE=−Dn^2logDn^2.

(6) To obtain a smooth SEE, the signal is passed through a zero-shift moving average filter [13] that can be considered as a moving window integrator. The mathematical expression of a moving average filter is expressed in the following where the window width is set as 33:

(8) $$\begin{array}{c} S_{n}^{S}=\frac{1}{N}\left\{S_{n-N/2}^{E}+\cdots +S_{n-1}^{E}+S_{n}^{E}+S_{n+1}^{E}+\cdots +S_{n+N/2}^{E}\right\}. \end{array}$$

The length of the moving average filter is an important parameter for this detection method. Generally, the length of the moving average filter is taken as approximately the width of the QRS complex. In the existing method [13], 65 samples are used for the moving average filter. However, in this paper, we reduce the length to 33.

### 2.3. Peak Energy Envelope Calculation

After the SEE calculation stage, we obtain the smooth peak signal, but it contains both true R peaks and false R peaks. In this stage, we thus attenuate the false R peaks and emphasize the true R peaks. This is performed by similar steps to those of the SEE, so we call this procedure the peak energy envelope (PEE).

(7) It is natural that the amplitude values for true R peaks are higher than those for false peaks. Thus, if we take the first-order differentiation of the signal, it stores the slope information of the true peaks but reduces the slope information of the false peaks. The first-order differentiation can be expressed as

(9) $$\begin{array}{c} D_{n}^{S}=S_{n+1}^{S}-S_{n}^{S}. \end{array}$$

(8) Next, the signal is amplitude normalized to unity as

(10) DnS^=DnSmax ∣ DnS ∣ .

(9) The normalized bipolar signal is converted to a unipolar signal by a squaring operation. The amplitude of false peaks is very low, and the squaring operation will attenuate these peaks completely. As a result, the true R peaks are amplified, and false R peaks are diminished. The squared unipolar signal can be expressed as:

(11) PnE=DnS^2.

(10) The signal is then passed through a moving average filter to obtain a resulting signal with smooth peak. In our proposed technique, we use a moving average filter length of 43 samples, which is smaller than the existing length of 85 [13]. The final signal expressed next is used for peak detection where the window width is set as 43.

(12) $$\begin{array}{c} P_{n}^{S}=\frac{1}{N}\left\{P_{n-N/2}^{E}+\cdots +P_{n-1}^{E}+P_{n}^{E}+P_{n+1}^{E}+\cdots +P_{n+N/2}^{E}\right\}. \end{array}$$

### 2.4. Peak Detection

**Figure 5:**
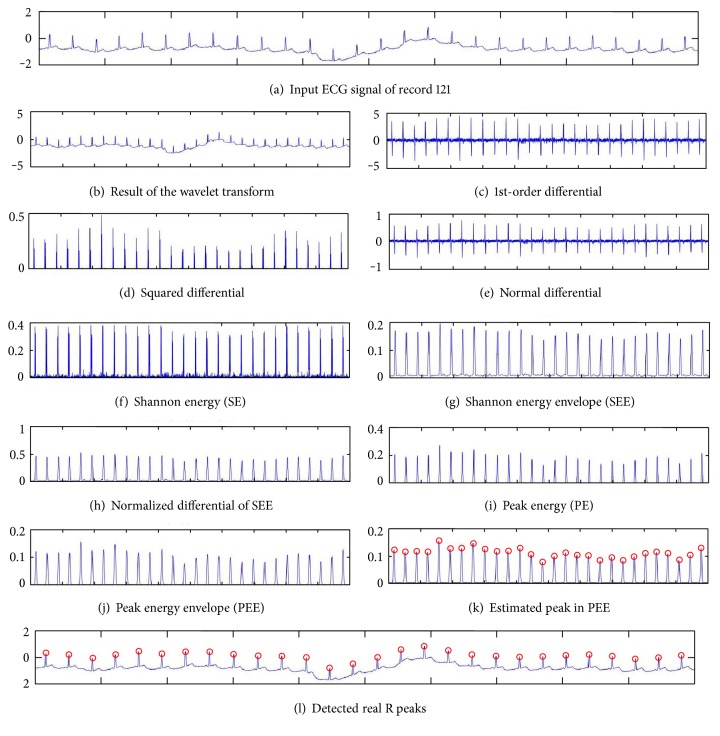
Example flow of the proposed R peak detection methods using WT and modified SEE. (a) Input ECG signal of record 121, (b) resized ECG after wavelet transform, (c) output of 1st-order differential, (d) squared differential, (e) normalized differential, (f) calculated Shannon energy (SE), (g) extracted Shannon energy envelope (SEE) after moving average, (h) normalized difference of SEE, (i) calculated peak energy (PE) after square operation, (j) extracted peak energy envelope (PEE) after moving average, (k) estimated peaks in PEE (red circles), and (l) true detected R peaks in ECG signal (red circles).

Figure 5 shows an example flow of the proposed WTSEE method for detecting R peaks. After squaring, to obtain a smooth SEE, a moving average filter operation is performed. The output of the moving average filter does not provide any unnecessary rising peaks.

(11) The locations of rising peaks are referred to as the locations of true R peaks, as shown in the Figure 5(k). Thus, no amplitude threshold value is required for the detection of R peaks.

(13) $$\begin{array}{c} \;R^{E}=findPeaks\left(P^{S}\right) \end{array}$$

where *R*^*E*^ is the estimated locations of rising peaks and findPeaks(^∗^) is a peak-finding function from smooth PEE *P*^*S*^.

(12) In this stage, the R peaks are detected by applying a peak-finding algorithm. However, the detected peak locations are slightly different from the actual positions of the R peaks in the ECG signal. Thus, to find the real positions of R peaks, the actual sample instant of R peaks in the input ECG signal is found by searching for the maximum amplitude within ±25 samples of the identified location in the previous step as

(14) $$\begin{array}{c} R_{k}^{C}=arg\underset{k}{max}\left\{E_{k-25}^{F},\ldots ,E_{k}^{F},\ldots ,E_{k+25}^{F}\right\}, \end{array}$$

where *R*_*k*_^*C*^ is the searched real position from the input ECG signal and *E*_*k*_^*F*^ is the amplitude of the *k*th positions of estimated R peak *R*_*k*_^*E*^.

In Figure 5, the total signal processing of the proposed method is shown, where red circles indicate the detected R peaks by using the proposed method in Figure 5(l).

### 2.5. R Peak Update

As the final procedure, the previously detected peak set *R*^*C*^ is validated and updated using the following steps. This validation and update process is based on the R-R intervals between neighboring R peaks. The objective of this procedure is balancing the R-R intervals.

(13) Measure the *x*-axis intervals between neighboring R peaks as follows:

(15) $$\begin{array}{c} ∆R^{C}\left(k\right)=x_{k+1}-x_{k},\;k=1,2,\ldots ,K-1. \end{array}$$

(14) Generate the final R feature, *R*^*F*^(*x*), by repeating the validation and update for all candidate peaks of *R*^*C*^(*x*). Each peak *R*^*C*^(*x*_*k*_) can be classified into one of three categories according to the value of the ∆*R*^*C*^(*k*):

-  *R*^*C*^(*x*_*k*_) is not included to *R*^*F*^(*x*) if the *x*-axis intervals between two neighboring peaks, ∆*R*^*C*^(*k* − 1) or ∆*R*^*C*^(*k*), are less than a given threshold; that is,

(16) $$\begin{array}{c} R^{C}\left(x_{k}\right)\notin R^{F}\left(x\right),\;if\;\Delta R^{C}\left(k-1\right)<\theta_{\Delta 1}\;or\;\Delta R^{C}\left(k\right)<\theta_{\Delta 1}, \end{array}$$

where *θ*_∆1_ is a threshold value that determines the minimum offset between R-R peaks. This threshold value can be set as follows:

(17) $$\begin{array}{c} \theta_{\Delta 1}=\alpha *\mu_{\Delta }, \end{array}$$

where *α* = 0.5 and *μ*_∆_ is the average spacing of R-R peaks.

(b) Find additional R peaks between *R*^*C*^(*x*_*k*−1_) and *R*^*C*^(*x*_*k*+1_) when *x*-axis intervals with two neighboring peaks are greater than a given threshold:

(18) $$\begin{array}{c} E^{F}\left(x_{a}\right)\in R^{F}\left(x\right),x_{k-1}+\gamma *\mu_{\Delta }<x_{a}<x_{k+1}-\gamma *\mu_{\Delta },if\;\Delta R^{C}\left(k-1\right)>\theta_{\Delta 2}\;or\;\Delta R^{C}\left(k\right)>\theta_{\Delta 2}, \end{array}$$

where *θ*_Δ2_ = *β*∗*μ*_Δ_ is a threshold value that determines the maximum interval between R-R peaks and *γ* is a parameter for defining the size of search area. We used *β* and *γ* as 1.5 and 0.5, respectively.

(c) *R*^*C*^(*x*_*k*_) is maintained as *R*^*F*^(*x*) when the *x*-axis intervals are located at the proper intervals:

(19) $$\begin{array}{c} {R^{C}\left(x_{k}\right)\in R^{F}\left(x\right),\;if\;\theta }_{∆1}\leq ∆R^{C}\left(k\right)\leq \theta_{∆2},\;\theta_{∆1}\leq ∆R^{C}\left(k+1\right)\leq \theta_{∆2}. \end{array}$$

## 3. Results

### 3.1. Experimental Data

We used the MIT-BIH arrhythmia database [28] from the PhysioNet site. This database was developed with the aim of benchmarking references for automated analysis of ECG signals. The MIT-BIH arrhythmia DB has 48 two-channel ambulatory ECG recordings of 30 min duration each. They are available at different frequencies and different time lengths. These signals are sampled at 360 samples per second and have a resolution of 11 bits over a 10 mV range. We used the first lead of all 48 records to validate the performance of the proposed method.

There are 112,647 labeled beats, where the “Normal beat” class contains 75,052 beats of annotation type “1.” Table 1 summarizes the annotations in the MIT-BIH arrhythmia DB and shows the examples of the 23 types of annotated beats. In the table, 1: NORMAL, 2: LBBB,…, and 38: PFUS are the annotation types and their abbreviations, respectively (please refer to the site for the meaning, annotation types, and their abbreviations). And # indicates the number of corresponding beats in MIT-BIH DB, and R-# is the record number of illustrated data.

As shown in Table 1, there are many abnormal beats in the ECG signals, so considering all annotations to be the reference peaks is not the correct way to verify the performance of the R peak detection methods. To solve the problem of incorrect or ambiguous annotation, we apply the validation process to determine whether each annotated position is true peak or not.

### 3.2. R Peak Detection Results

**Figure 6:**
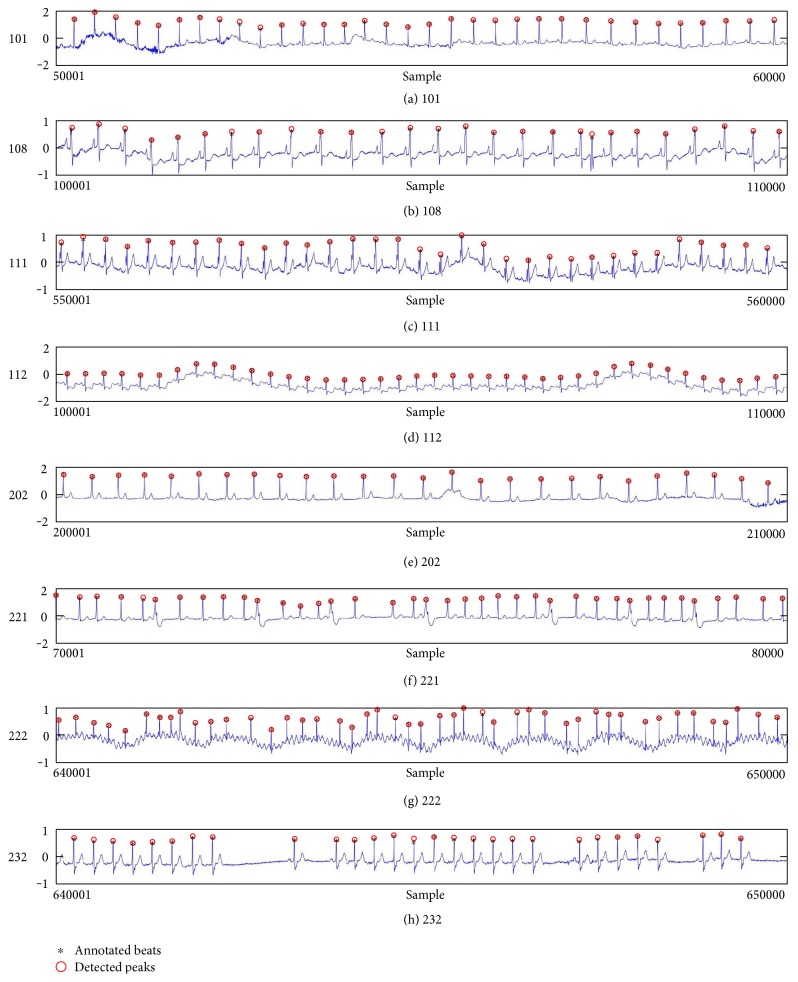
Examples of the detected R peaks from various normal records: (a) 101, (b) 108, (c) 111, (d) 112, (e) 202, (f) 221, (g) 222, and (h) 232. In the figure, black asterisks (^∗^) denote the annotated beats in MIT DB and red circles (O) denote the extracted R peaks.

Figure 6 shows examples of the detected R peaks from various records in the MIT-BIH DB. In this figure, black asterisks (^∗^) denote the annotated beats in DB and red circles (O) denote the extracted peaks. As shown in the figure, the proposed method can detect normal R peaks under various conditions such as baseline drift, noisy signal, tall T waves as shown in Figure 6(c), or long paused waves as shown in Figure 6(h).

**Figure 7:**
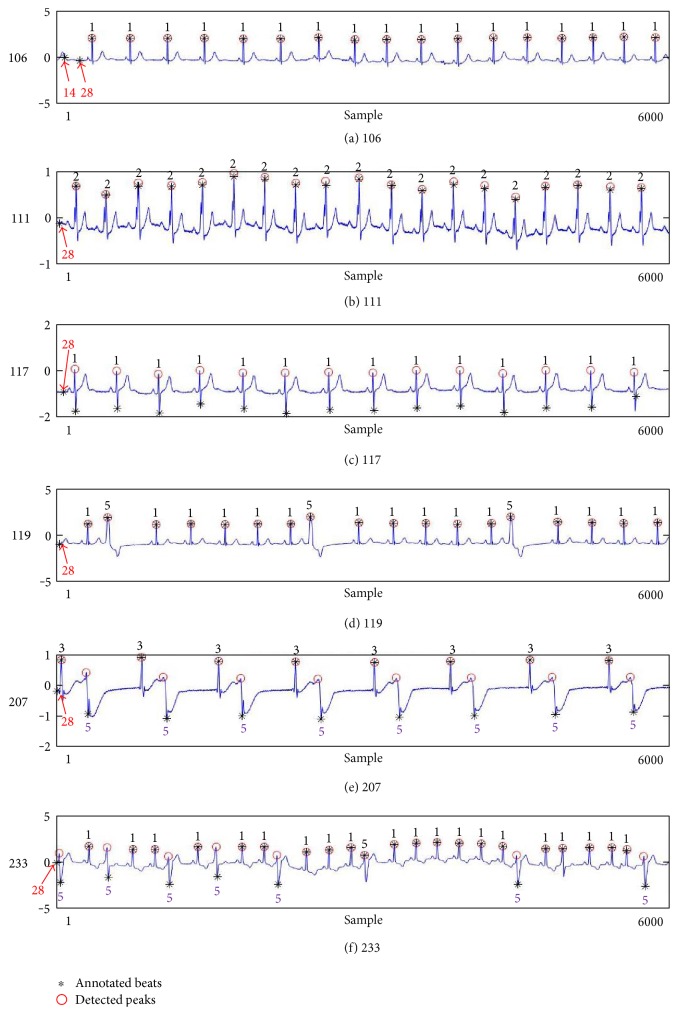
Examples of the annotated beats and detected real peaks from various records: (a) 106, (b) 111, (c) 117, (d) 119, (e) 207, and (f) 233. In the figure, the numbers such as 1, 2, 3, 5, 14, and 18 indicate the annotation types of each beat.

Figure 7 shows another example of the detected R peaks and annotated beats from various records in the MIT-BIH database. In this figure, numbers, such as 1, 2, 3, 5, 14, and 28, indicate the annotation types of each beat. As shown in the figure, the proposed method can remove abnormal beats at the initial position of the ECG signal and correct the location errors of record 117 as shown in Figure 7(c).

**Figure 8:**
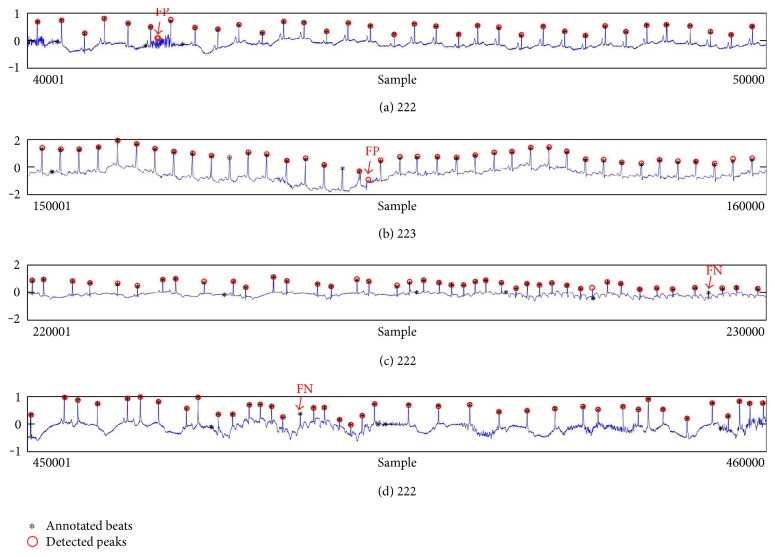
Detection failure of the proposed WTSEE method.

Despite the superiority of the proposed method, detection errors still exist.  Figure 8 demonstrates examples of false positives (FP) and false negatives (FN) of the proposed method. FP is mainly generated in a high-frequency region, and FN occurs in a small-noise area between R peaks. Especially, the DER of the proposed method appears the best among others.

### 3.3. Evaluation Measure and Results

To evaluate the performance of our proposed R peak detection method, we require three parameters—namely, true positive (TP), false negative (FN), and false positive (FP)—from the detected R peak. Here, TP is the number of correctly detected R peaks, FN is the number of missed R peaks, and FP is the number of noise spikes incorrectly detected as R peaks. Sensitivity (Se), positive predictability (+P), detection error rate (DER), and accuracy (Acc) can be computed by using TP, FN, and FP by using the following equations, respectively, as widely used in the literatures [1, 2, 4, 13, 24–26]:

(20) $$\begin{array}{c} Se=\frac{TP}{TP+FN}\times 100\%,\\ +P=\frac{TP}{TP+FP}\times 100\%,\\ DER=\frac{FP+FN}{TP}\times 100\%,\\ Acc=\frac{TP}{TP+FP+FN}\times 100\%, \end{array}$$

where TP are the correctly detected beats (true positive), FP are falsely detected beats (false positive), and FN are the undetected beats (false negative). The sensitivity is used for evaluating the ability of the algorithm to detect true beats, the positive predictability is used for evaluating the ability of the algorithm to discriminate between true and false beats, and the error rate is used for evaluating the accuracy of the algorithm [25].

The performance of the proposed R peak detection method for 48 ECG recordings of the MIT-BIH arrhythmia database is summarized in Table 2. The proposed method detects a total of 109,415 (In total, there are 116,137 annotated beats in MIT-BIH DB, but this number varies among different references due to the use of different steps and comparing tools [24]) true peaks. It also produces 79 FNs and 99 FPs. The average accuracy for the proposed method is 99.838%, the sensitivity is 99.93%, the positive predictability is 99.91%, and the detection error rate is 0.163%. The obtained performance is comparable to the best performances reported in the literature considering the processing speed and memory use. The high detection accuracy of the proposed method is especially meaningful because it is based on wavelet transform. Previous studies using wavelet transform did not show high performance compared with nonwavelet transform-based methods. Moreover, the final validation procedure proposed in our method verifies the validity of each detected peak and further improved the peak detection accuracy.

In Table 3, the performance of the proposed method on the MIT-BIH arrhythmia DB is compared with other existing methods. It shows that our proposed method has comparable accuracy to other conventionally used methods including wavelet transform techniques, the differential operation method, the Pan and Tompkins algorithm, SEHT, and the Shannon energy technique.

The previous four measures are related only to the R peaks. Recently, measures of HRV have been suggested, such as the mean of R-R intervals (MRR), standard deviation of normal to normal R-R intervals (SDNN), and root mean square of normal to normal RR intervals (RMSSD), where R is the peak of a QRS complex (heartbeat) [29].

- MRR is measured by

(21) $$\begin{array}{c} MRR=\overset{-}{I}=\frac{1}{N-1}\overset{N}{\underset{n=2}{\sum }}I\left(n\right), \end{array}$$

where *N* is the total heart beats (*N* = TP + FN), *I*(*n*) is R-R interval between *n*th R peak and previous R peak, and $\overset{-}{I}$ is a mean of R-R intervals.

(ii) SDNN is defined by

(22) $$\begin{array}{c} \;SDNN=\sqrt{{\frac{1}{N-1}\overset{N}{\underset{n=2}{\sum }}\left[I\left(n\right)-\overset{-}{I}\right]}^{2}}. \end{array}$$

(iii) RMSSD is measured by

(23) $$\begin{array}{c} \;RMSSD=\sqrt{{\frac{1}{N-2}\overset{N}{\underset{n=3}{\sum }}\left[I\left(n\right)-I\left(n-1\right)\right]}^{2}}. \end{array}$$

**Figure 9:**
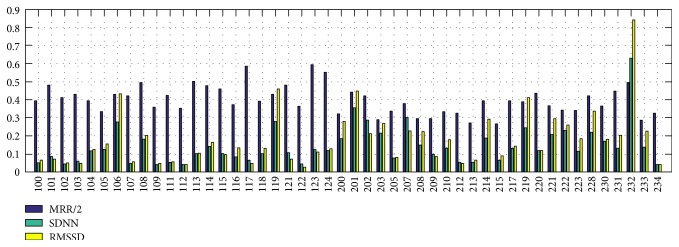
HRV measurements of the MIT-BIH DB. MRR: mean R-R intervals (ms), SDNN: standard deviation of normal to normal R-R intervals, RMSSD: root mean square of normal to normal R-R intervals.

Figure 9 depicts the measured MMR, SDNN, and RMSSD from R-R intervals of the extracted R peaks. By investigating these measures, initial diagnosis of CVDs for patients can be achieved. In Figure 9, half of MRRs are illustrated to show three different kinds of measures together. The proposed method obtained MRR of 0.808, SDNN of 0.145, and RMSSD of 0.193, on average.

### 3.4. Experimental Environment and Real-Time Implementation

The proposed R peak detection method using WT and modified SEE has been implemented with MATLAB R2016b on a PC with a 3.3-GHz Intel Core i5-4590 CPU, 4 GB of memory on Windows 7. The average processing time of our method is approximately 13.7 s on 48 ECG records in the MIT-BIH arrhythmia DB with a length of 30 minutes. As a result, the average throughput is 28.6 ms for an ECG signal. We showed exact parameter values used in our experiments around each of the equations throughout the manuscript to help researchers who want to implement our methods. It is recommended that researchers carefully follow the descriptions provided in Section 2.5 to obtain the same peak detection accuracies as those presented in this paper.

### 3.5. Discussions

This study presented our approach using WT and modified SEE for detecting R peaks of an ECG signal in the QRS complex. We used WT to take advantage of its representation power of temporal features at various resolutions. We applied WT to ECG signals to perform both down-sampling and noise reduction in a single step. This WT replaces the band-pass filtering used in a few representative ECG signal analysis methods [13, 25]. We also applied a soft-thresholding method for noise removal with keeping informative signals. The application of SEE and PEE can be considered as similar to the methods in [13]. However, we introduced additional postprocessing steps (Section 2.5) where the R-R peak intervals were inspected to determine whether to keep each detected R peak or detect additional R peaks in an R-R peak interval.

The novelty of our work is twofold: (1) Firstly, we proposed an ECG analysis technique that starts with WT method. There have been many WT-based methods, but their performances have been observed lower than those of non-WT-based methods [1, 4, 10, 13, 16–18, 25]. Our study demonstrates the possibility of obtaining high-peak detection accuracy in ECG signal analysis by using WT method. (2) Secondly, we proposed a postprocessing method that verifies the validity of each detected peak and indicates the probability of a missing peak.

The experimental results of our study show reasonable R peak detection performance with using less memory and processing time. This study has diverse applications in the initial diagnosis of CVDs for remote patients. The quantitative parameters (i.e., sensitivity, positive predictability, detection error rate, and accuracy) confirm acceptable performance of the proposed WTSEE detection method in practical applications.

## 4. Conclusion

In this paper, we presented a novel approach to detecting R peaks of an ECG signal and calculating the R-R interval of the R peaks via the wavelet transform (WT) method and a modified Shannon energy envelope (SEE) for rapid ECG analysis. The proposed method utilized WT and showed superior performance to other WT-based methods. We believe that the use of WT combined with subsequent processing steps properly analyzed the ECG signals to detect R peaks with high accuracy. Moreover, the proposed postprocessing method effectively validated each detected peak and indicated miss detection to further improve the peak detection accuracy.

The proposed system was tested using the MIT-BIH arrhythmia DB. We analyzed the system performance for sensitivity, positive predictability, detection error rate, and accuracy. We compared the proposed approach with the existing techniques to validate the superior performance of the former. In the future, we would like to apply our analysis method to detecting other peak points in ECG signals. Detecting various types of peaks in ECG signal will provide more useful information for diagnosing CVDs and create a number of related applications.

## Figures and Tables

**Table 1:** Summary and example of the 23 types of annotated beats in the MIT-BIH arrhythmia DB.

1: NORMAL (75,025)	2: LBBB (8075)	3: RBBB (7529)	4: ABERR (150)
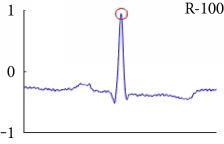	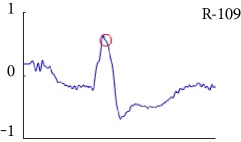	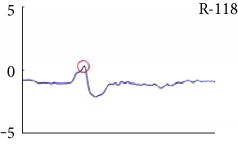	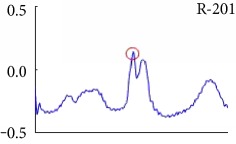
5: PVC (7130)	6: FUSION (803)	7: NPC (83)	8: APC (2546)
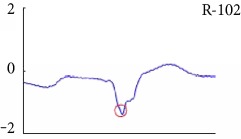	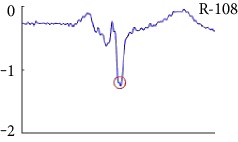	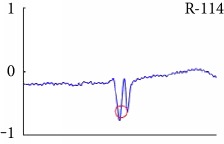	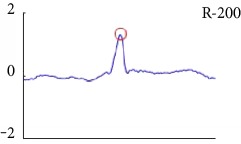
9: SVPB (2)	10: VESC (106)	11: NESC (229)	12: PACE (7028)
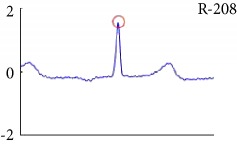	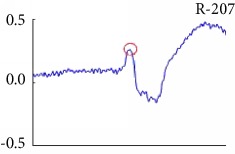	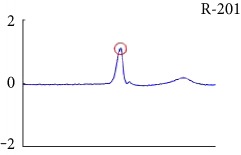	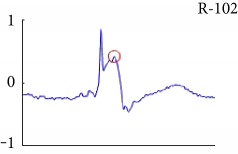
13: UNKNOWN (33)	14: NOISE (616)	16: ARFCT (132)	22: NOTE (437)
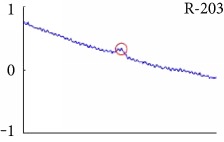	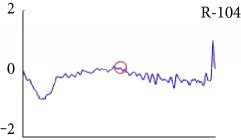	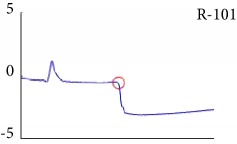	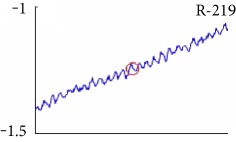
28: RHYTHM (1291)	31: FLWAV (472)	32: VFON (6)	33: VFOFF (6)
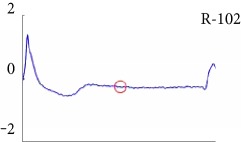	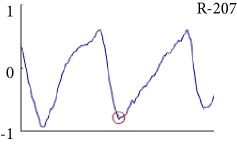	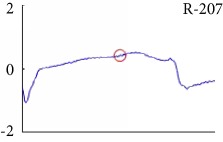	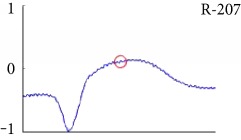
34: AESC (16)	37: NAPC (193)	38: PFUS (982)	23 types of annotation Total annotated beats: 112,647
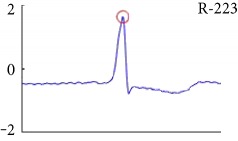	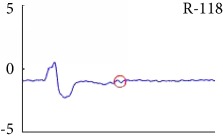	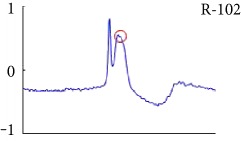	

**Table 2:** Performance of the proposed R peak detection method using the MIT-BIH arrhythmia DB with length 30 min.

Record	Total beats (TP + FN)	TP (beats)	FN (beats)	FP (beats)	Se (%)	+P (%)	DER (%)	Acc (%)
100	2273	2272	1	0	99.96	100	0.044	99.956
101	1865	1864	1	3	99.95	99.84	0.215	99.786
102	2187	2187	0	0	100	100	0	100
103	2084	2084	0	0	100	100	0	100
104	2229	2228	1	5	99.96	99.78	0.269	99.731
105	2572	2568	4	17	99.84	99.34	0.818	99.189
106	2027	2024	3	5	99.85	99.75	0.395	99.606
107	2137	2136	1	1	99.95	99.95	0.094	99.906
108	1763	1759	4	5	99.77	99.72	0.512	99.491
109	2532	2532	0	1	100	99.96	0.039	99.961
111	2124	2123	1	0	99.95	100	0.047	99.953
112	2539	2539	0	0	100	100	0.000	100.000
113	1795	1794	1	0	99.94	100	0.056	99.944
114	1879	1878	1	2	99.95	99.89	0.160	99.841
115	1953	1953	0	3	100	99.85	0.154	99.847
116	2412	2400	12	2	99.50	99.92	0.583	99.420
117	1535	1532	3	1	99.80	99.93	0.261	99.740
118	2278	2278	0	2	100	99.91	0.088	99.912
119	1987	1986	1	2	99.95	99.90	0.151	99.849
121	1863	1862	1	1	99.95	99.95	0.107	99.893
122	2476	2476	0	0	100	100	0	100
123	1518	1518	0	2	100	99.87	0.132	99.868
124	1619	1619	0	4	100	99.75	0.247	99.754
200	2601	2600	1	2	99.96	99.92	0.115	99.885
201	1963	1960	3	0	99.85	100	0.153	99.847
202	2136	2135	1	0	99.95	100	0.047	99.953
203	2980	2959	21	8	99.30	99.73	0.980	99.029
205	2656	2655	1	0	99.96	100	0.038	99.962
207	1860	1858	2	5	99.89	99.73	0.377	99.625
208	2955	2946	9	3	99.70	99.90	0.407	99.594
209	3005	3005	0	1	100	99.97	0.033	99.967
210	2650	2649	1	2	99.96	99.92	0.113	99.887
212	2748	2748	0	0	100	100	0	100
213	3251	3251	0	0	100	100	0	100
214	2262	2262	0	1	100	99.96	0.044	99.956
215	3363	3362	1	0	99.97	100	0.030	99.970
217	2208	2208	0	1	100	99.95	0.045	99.955
219	2154	2154	0	0	100	100	0	100
220	2048	2048	0	0	100	100	0	100
221	2427	2427	0	1	100	99.96	0.041	99.959
222	2483	2483	0	1	100	99.96	0.040	99.960
223	2605	2605	0	0	100	100	0	100
228	2053	2052	1	12	99.95	99.42	0.634	99.370
230	2256	2256	0	2	100	99.91	0.089	99.911
231	1571	1571	0	0	100	100	0	100
232	1780	1780	0	2	100	99.89	0.112	99.888
233	3079	3076	3	2	99.90	100	0.163	99.838
234	2753	2753	0	0	100	100	0	100
Total	109,494	109,415	79	99	99.93	99.91	0.163	99.838

**Table 3:** Comparison of the proposed WTSEE method with other methods for detecting R peaks using the MIT-BIH database.

Method	Total beats^a^ (TP + FN)	TP (beats)	FN (beats)	FP (beats)	Se (%)^b^	+P (%)^b^	DER (%)^b^	Acc (%)^b^
Proposed WTSEE	109,494	109,415	79	99	99.93	99.91	**0.163**	99.838
ISEE, 2016 [13]	109,532	109,474	**58**	116	**99.95**	99.89	0.159	**99.841**
PSEE, 2013 [23]	109,494	109,401	93	**91**	99.92	**99.92**	0.168	99.832
SEHT, 2012 [22]	109,496	109,417	79	140	99.93	99.87	0.200	99.800
DOM, 2008 [12]	109,809	109,751	**58**	166	**99.95**	99.85	0.204	99.796
STSE, 2014 [24]	108,494	108,323	171	97	99.84	99.91	0.247	99.753
WT, 2004 [16]	109,428	109,208	220	153	99.80	99.86	0.342	99.660
PT, 1985 [14]	109,809	109,302	507	277	99.54	99.75	0.717	99.288

^a^Total beats—the sum of total beats of all analyzed records in each study. ^b^These values are calculated by total beats.
